# Comparison of the Effects of Conching Parameters on the Contents of Three Dominant Flavan3-ols, Rheological Properties and Sensory Quality in Chocolate Milk Mass Based on Liquor from Unroasted Cocoa Beans

**DOI:** 10.3390/molecules26092502

**Published:** 2021-04-25

**Authors:** Bogumiła Urbańska, Hanna Kowalska, Karolina Szulc, Małgorzata Ziarno, Irina Pochitskaya, Jolanta Kowalska

**Affiliations:** 1 Department of Technology and Food Evaluation, Institute of Food Sciences, Warsaw University of Life Sciences, 159c Nowoursynowska St., 02-776 Warsaw, Poland; malgorzata_ziarno@sggw.edu.pl (M.Z.); jolanta_kowalska@sggw.pl (J.K.); 2 Department of Food Engineering and Process Management, Institute of Food Sciences, Warsaw University of Life Sciences, 159c Nowoursynowska St., 02-776 Warsaw, Poland; hanna_kowalska@sggw.edu.pl (H.K.); karolina_szulc@sggw.edu.pl (K.S.); 3 The Scientific and Practical Centre for Foodstuffs of the National Academy of Sciences of Belarus, 29. Kozlova St., 220037 Minsk, Belarus; pochitskaja@yandex.ru

**Keywords:** conching, milk chocolate, catechin, epicatechin, procyanidin

## Abstract

The content of polyphenols in chocolate depends on many factors related to the properties of raw material and manufacturing parameters. The trend toward developing chocolates made from unroasted cocoa beans encourages research in this area. In addition, modern customers attach great importance to how the food they consume benefits their bodies. One such benefit that consumers value is the preservation of natural antioxidant compounds in food products (e.g., polyphenols). Therefore, in our study we attempted to determine the relationship between variable parameters at the conching stage (i.e., temperature and time of) and the content of dominant polyphenols (i.e.,catechins, epicatechins, and procyanidin B2) in chocolate milk mass (CMM) obtained from unroasted cocoa beans. Increasing the conching temperature from 50 to 60 °C decreased the content of three basic flavan-3-ols. The highest number of these compounds was determined when the process was carried out at 50 °C. However, the time that caused the least degradation of these compounds differed. For catechin, it was 2 h; for epicatechin it was 1 h; and for procyanidin it was 3 h. The influence of both the temperature and conching time on the rheological properties of chocolate milk mass was demonstrated. At 50 °C, the viscosity and the yield stress of the conched mass showed its highest value.

## 1. Introduction

The cocoa bean (*Theobroma cacao* L.) and its processing products (cocoa liquor, cocoa powder, and chocolate) are rich sources of bioactive compounds, including polyphenols. Polyphenols are secondary plant metabolites that belong to a subclass of a larger group of plant compounds known as flavonoids [1]. Many studies have shown that consumption of chocolate and cocoa powder (rich in flavonoids) reduces the risk of cardiovascular disease [1,2,3,4,5].

Polyphenol content accounts for about 12–18% of a cocoa bean’s dry weight. About 60% of the total polyphenol content in unfermented cocoa beans are monomeric and oligomeric flavanols. The major monomeric flavanol is (−)-epicatechin (making up to 35% of the total polyphenol content), followed by (+)-catechin and procyanidin B2 (epicatechin- (4β-8)-epicatechin) [1,6]. Cocoa and chocolate are among the most concentrated sources of the flavonoids catechin, epicatechin, and procyanidin [7].

The basic ingredients needed to make chocolate are cocoa liquor, sugar (or other sweeteners), and cocoa butter (cocoa fat). Emulsifiers, flavors, cocoa powder, and other ingredients such as nuts can also be added. Milk chocolates, according to Directive 2000/36/EC [8], should not contain less than 25% cocoa solids nor less than 14% milk solids.

The polyphenols are present in the fat-free components of cocoa beans. The content of these compounds can vary considerably depending on the variety of bean, growing conditions, processing technologies, and their parameters [9]. Conching is an important stage for improving the quality of chocolate. Its main purpose is to thoroughly combine all ingredients to obtain homogeneous mass [10]. Moreover, during conching, unwanted volatile compounds (e.g., acetic acid) and water are evaporated, shaping the color, texture, and flow characteristics. The amount of moisture removed varies depending on the raw material in different types of chocolate [10]. Manufacturers use different temperatures and conching times for chocolate mass depending on the variety of cocoa beans used and their origin, its raw material composition, and the expected properties of the final product (the type of chocolate) [11]. Bolenz et al. [12] found that the flavor at the conching stage of milk chocolate is less important than dark chocolate, which reduces costs. This suggests the omission of dry conching and modification of raw material composition.

Temperature and stirring used during conching are important for the formation and intensity of Maillard reaction and Strecker degradation [13]. However, these parameters vary depending on the type and composition of the product. For example, in milk chocolate, conching temperatures below 50 °C are preferred to avoid the formation of Maillard compounds [10,14]. Conching can be described as a high-cost process due to the long duration maintenance of high temperatures, which amounts to many hours [15,16,17]. The time required for this process mainly depends on the amount of material and the configuration of the conching equipment [16].

One of the issues to be considered in short conching is the water vapor absorbed from the air by the main chocolate ingredients. During refining, water passes from the milk powder and cocoa liquor to the amorphous surface of the crushed sugar particles, where it forms a solvated layer of sugar dissolved in water that can cause the particles to clump together [18]. The unwanted moisture should be removed in the dry conching stage to disperse the agglomerates. Too much water content in the chocolate mass can lead to efflorescence, mainly sugar efflorescence, so it is important that water content in the product is <1.0%, and preferably 0.4–0.6% [18,19,20]. In addition, shortening the conching time should consider the quality and properties of the cocoa liquor. With the development of cocoa processing technology and high-quality varieties, cocoa liquor with desired flavor quality, low acidity, and moisture content can be obtained. This situation is advantageous for bypassing or shortening the dry phase at the conching stage [19].

There are a limited number of studies on the change in composition and levels of phenolic compounds during conching. Schumacher et al. [21] showed that conching had no effect on the polyphenol content of chocolates. However, Sulistyowati and Misnawi [22] found that polyphenol concentration, especially simple phenols, and antioxidant activity decreased significantly after the conching process. Di Mattia et al. [23] determined changes in the composition of phenolic compounds as a function of time and conching conditions. They also found that short conched chocolates have a higher content of phenolic monomers.

Chocolate quality is determined during the manufacturing process and can be monitored by variables such as viscosity, texture, and moisture content, which are factors that influence sensory attributes [24,25]. Viscosity is important for handling chocolate mass during production (e.g., during pumping and mixing) because it determines its suitability for the product’s end use, i.e., enrobing or dipping [26]. To determine the effect of conching conditions on the concentration and profile of important flavanols (i.e., catechins, epicatechins, and procyanidins), rheological properties, and the sensory quality of chocolate mass, the present study was carried out to consider the variable parameters of the process (time and temperature).

## 2. Results and Discussion

Here, we characterized and quantified three major phenolic compounds of the flavan-3-ol class in 9 CMM (chocolate milk mass) using high performance liquid chromatography analysis. The results of all determinations are presented in Table 1. In addition, the ratio of epicatechin to catechin (epi/cat) for all CMM samples is shown in Table 1.

**Figure 1:**
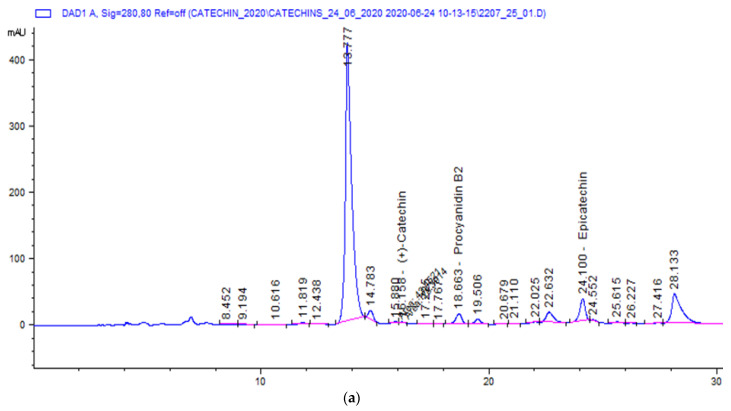

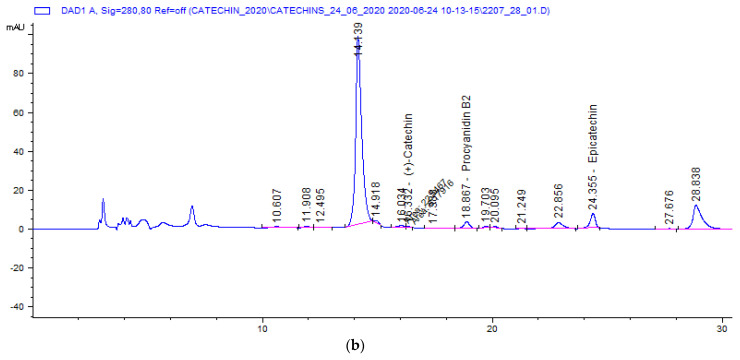
(**a**) Exemplary chromatogram profile of cocoa liquor from Peru. (**b**) Exemplary chromatogram profile of CMM (C503h).

Figure 1 shows an exemplary chromatographic profile for the contents of three compound groups of flavanols that were determined in the investigated CMM.

In this study, the contents of three compounds groups from the flavanol class were evaluated: (+)-catechin, (−)-epicatechin, and procyanidin B2 (Table 1). These compounds were selected due to the fact that, according to the literature, they are the predominant representatives of the phenolic substance class in cocoa beans. Flavan-3-ols are the main class of flavonoids detected in cocoa beans and account for about 92% of all phenolic compounds [27]. The epi/cat ratio presented in Table 1 is a widely used indicator because it can be related to the degree of cocoa processing [28,29,30]. It is assumed that as temperature increases, the epi/cat ratio tends to decrease due to isomerization reactions and faster degradation of epicatechin relative to catechin [31]. According to Hurst et al. [32], high temperatures can induce epimerization of epicatechin to (−)-catechin and (+)-catechin to (+)-epicatechin. Racine et al. [33] showed that the bean’s fermentation process causes losses of (−)-epicatechin (77–94%) and (+)-catechin (83–89%), depending on its origin and variety. Moreover, for the degree of polymerization for the total flavanols, the reduction in their content was 61% for monomers and 54% for dimers. Although the loss of cocoa polyphenols may reduce the positive impact on the overall health benefits of cocoa consumption, it is important to understand that these losses correlate with the development of a positive sensory profile of raw material. According to Aprotosoaie et al. [34], (−)-epicatechin accounts for up to 35% of the total polyphenolic content in cocoa. Similar proportions of individual flavanols were obtained by Oracz et al. [27] in a quantitative–qualitative analysis of polyphenols in cocoa beans (regardless of seed variety, e.g., Forastero, Nacional, and Trinitario from Brazil, Ecuador, and Venezuela, respectively). It should also be noted that the differences between the results presented in the literature may be due to the method of determination used, as well as the presentation of the final results (their conversion, e.g., total flavan-3-ols converted to (+)-catechin).

### 2.1. Determination of the Content of Dominant Flavanols in Raw Materials Used to Produce CMM

A study by Kofink et al. [35] showed that roasted cocoa beans and cocoa products can contain flavan-3-ol (−)-catechin along with (+)-catechin and (−)-epicatechin. Degradation of (−)-epicatechin has been observed during manufacturing. Maintaining a high temperature while roasting cocoa beans (especially for the alkalization of cocoa powder) triggers the epimerization reaction. This caused the level of (−)-epicatechin to decrease to (−)-catechin. A typical RP-HPLC analysis is limited in separating enantiomers of catechin and epicatechin [9]. According to Kofink et al. [35] (+)-catechin is only a minor component of the natural flavanols in cocoa, which contrasts with the dominant (−)-epicatechin. Consequently, only small amounts of (+)-catechin form during the epimerization reaction, at least when compared to (−)-epicatechin levels [35].

#### 2.1.1. Catechin Content in Raw Materials

In the analyzed cocoa beans, the catechin content was approximately 13.56 mg/100 g. In cocoa liquor, it was approximately 11.61 mg/100 g (Table 2). Peláez et al. [36] analyzed polyphenols present in cocoa and showed catechin contents of 65 mg/100 g for fresh cocoa beans and 53, 27, and 16 mg for beans fermented for 48, 96, and 120 h, respectively. These results are significantly higher than those obtained in this study, which is related to the longer duration of fermenting cocoa beans. In a study by Ioannon et al. [37], raw cocoa beans were not subjected to roasting, and thus the catechin content was 134 mg/100 g during roasting. At 145 °C it degraded to 52 mg/100 g. Similar results were obtained by Jolić et al. [38], Kothe et al. [39], and Oracz et al. [27], whose studies focused on the changes in the qualitative characteristics of flavanols present in cocoa beans at different processing stages, with particular emphasis on the roasting stage. In a study by Oracz et al. [27], the catechin content of roasted cocoa beans (under different process parameters) varied significantly and ranged from 2 to 105 mg/100 g dw. The increase in catechin levels during high temperature processing may be due to the epimerization of (−)-epicatechin (2R, 3R) into its epimer, (−)-catechin (2S, 3R) [35]. The higher concentration of catechins can also be attributed to the degradation of procyanidins (under the action of high temperature at the roasting stage) to their free flavan-3-ol monomer units, such as (+)-catechin and (−)-epicatechin [40]. 

Oracz et al. [27] found a significant increase in catechin content at 110 °C in most types of cocoa beans, except for those grown in Ecuador (Trinitario), Papua New Guinea, and Indonesia. They also found that catechin concentration increased faster when there was limited oxygen access to the roasted material. Similarly, Wang and Helliwell [40] noted that, at high temperatures, especially under anaerobic conditions, more catechins, which are (−) epiphorms (2R, 3R), could be converted to their respective (−) forms (2S, 3R). Similar conclusions were reached by Tamrin et al. [41], who reported that roasting cocoa powder in a roasting chamber under a vacuum (45.6 and 60.8 cm Hg) at 100 to 120 °C with low oxygen levels can slow down the oxidation processes of catechins. Oracz et al. [27] found the greatest increase in catechin content when cocoa beans were roasted at 135 and 150 °C, using an air humidity of RH = 5.0%. After roasting under these conditions, catechin concentrations in roasted cocoa beans were about 2 to 12 times higher tha raw samples. The observed changes in catechin levels during roasting varied widely. Kendari et al. [42] showed that roasting under near-vacuum conditions can slow down the catechin oxidation processes. Epimerization may also result from the application of cocoa alkalization due to the synergistic effect of elevated temperatures and significant changes in the raw material’s pH [43]. In a study by Stanley et al. [44], roasting cocoa beans at 150 °C or higher increased the levels of catechin heptamers and heptamers. The presented research results indicate that the influence of temperatures on the content of phenolic compounds lack a clear influence as to how temperature impacts individual enantiomers. Due to the limited research on the conching process’ influence, studies have been initiated on other thermal processes that accompany cocoa bean processing.

#### 2.1.2. Epicatechin Content in Raw Materials

The study showed that initial unroasted cocoa beans contained 226.65 mg/100 g of epicatechin, while in beans used for production it was 261.34 mg/100 g (Table 2). The obtained results are similar to the data presented by other authors (e.g., Oracz et al. [27], who reported that among the flavan-3-ols in roasted cocoa bean samples, the highest amount of epicatechin was determined; its concentration, depending on the groups, ranged from 37 to 872 mg/100 g dw). A rapid decrease in epicatechin content was found when the roasting temperature increased from 110 to 150 °C. The changes in the content of individual flavan-3-ols observed in roasted cocoa beans may be due to the oxidation or degradation of these compounds during high-temperature processing [40,45]. Flavan-3-ol monomers may participate in coupled oxidation reactions followed by polymerization and condensation of oxidized derivatives with other phenolic compounds or proteins, leading to complex high-molecular weight structures [46]. According to Kofin et al. [35], epicatechin may be degraded by epimerization reactions that occur under roasting conditions. Oracz et al. [27] roasted at 110 and 120 °C, which was compared to higher temperatures that resulted in better stability of epicatechin. It is worth noting that the loss of this compound also depends on the roasting moisture content. Therefore, epicatechin levels changed the least when roasted at 110 °C with RH = 5.0%. The roasting humidity (RH) increased with a saturated steam (pressure 0.2 MPa), which was obtained via a steam generator. This behavior can be explained by a protective barrier that formed around the beans via moisture. This then limited oxygen diffusion into the seeds. Similar conclusions were reached by Zzaman et al. [45], who hypothesized that the absence of oxygen reduced flavonoid loss during roasting with superheated steam compared to convection roasting at the same temperature and time. 

In this study, at 150 °C, the greatest decrease in epicatechin content was observed when RH was 2.0%. Heat treatment under these conditions resulted in the loss of epicatechin, which ranged from 53 to 75% of the initial levels in raw cocoa beans. This may be related to the formation of free space between the shell and kernel during the processing of cocoa beans at high temperatures and increased humidity. Consequently, the heat transfer inside the kernel is higher and the kernel temperature increases rapidly, leading to undesirable changes due to oxidation [38,39]. In general, the changes in all cocoa groups showed a similar trend. The lowest reduction of epicatechin was found in roasted Forastero beans from Brazil; the highest was found in bean samples from Ghana. A study by Kim et al. [47] confirmed the differences in epicatechin and other phenolic compounds, depending on the variety and growing region. Researchers showed that there was a six-fold variation in epicatechin content in fermented cocoa beans from different regions. Ioannona et al. [37] analyzed the effect of temperature on the content of phenolic compounds. They determined the content of epicatechin in unroasted beans at 112 mg/100 g. In beans roasted at 145 °C, it was more than five times less (21 mg/100 g). Moreover, the authors proposed to use the ratio of epicatechin to catechin content as an indicator of changes that occurred during high-temperature processes. Similar to Caligiani et al. [31], they showed that, as a result of high temperature, the degradation of epicatechin is faster than catechin, which is likely due to the isomerization of epicatechin to (+) catechin. In our study, the ratio of epicatechin to catechin was 19.27 and 19.52 in unroasted beans and cocoa pulp, respectively, thus indicating a low degree of processing raw cocoa materials. The results are consistent with the data presented by Payne et al. [30], who obtained an epi/cat ratio value of 20.1 for dry fermented cocoa beans before the roasting process.

#### 2.1.3. Procyanidin B2 Content in Raw Materials

The procyanidin B2 content of cocoa liquor from roasted beans was 93.0 mg/100 g and that of unroasted beans was 107.13 mg/100 g (Table 2). Pettipher et al. [48] showed that procyanidins are converted to insoluble structures with a reddish-brown color, resulting in the characteristic coloring of chocolate during fermentation and roasting. Therefore, numerous studies have described the loss of compounds in cocoa beans during these processes. Tomas-Barberán et al. [49] studied the development of a procedure that obtains cocoa powder with increased flavanols content. They obtained a product characterized by nine-times higher procyanidin B2 content (2430 mg/100 g) than traditional cocoa (262 mg/100 g). This was the result of the selection of cocoa beans and the application of special processing, which corresponded to the patent obtained by the Callebaut brand in 2009 and 2015. 

The results obtained in this study were within the lower limit of procyanidin content as determined by Oracz et al. [27] in cocoa beans roasted under varying conditions of temperature and air access. Compared with unroasted cocoa beans, roasting caused a significant (*p* < 0.05) decrease in procyanidin B2 levels (3–72%). This behavior can be explained by the fact that procyanidins readily degrade during heat treatment in the presence of oxygen to their monomeric units or bind to other phenolic compounds, proteins, polysaccharides, and alkaloids, as well as Maillard reaction products. This leads to the formation of molecular complexes [50]. Oracz et al. [27] also found that procyanidin losses showed a similar trend to epicatechin and were lower when cocoa beans were roasted at 110 °C. Heat treatment at 150 °C was the most destructive. The results further indicate that the stability of procyanidins in cocoa beans is strongly dependent on cocoa bean properties. 

In the quoted studies, the value of procyanidin B2 in different types of beans ranged from 41–425 mg/100 g. The progressive decrease in phenolic substances, including procyanidins, during chocolate production was also described by Di Mattia et al. [51], who indicated that roasting plays the most important role. Lemarcq et al. [52] showed that the level of procyanidin B2 did not significantly change in liquor obtained from beans roasted at 130 °C for 30 min. Its concentration decreased significantly when a more intense roasting treatment was applied, with the greatest effect for the sample processed at 160 °C for 30 min. The use of such parameters resulted in a reduction of procyanidin B2 by almost 70%, which was compared to the unroasted bean sample. Jolić et al. [38] reported that not only the roasting process affects changed the loss of procyanidins but also alkalization. The procyanidin B2 content shown in the study was 63 mg/100 g in the raw starting (fermented and dried) beans, 44 mg/100 g in roasted beans, and more than four-times less (14 mg/100 g) in cocoa beans obtained using both operations (Table 2). Di Mattia et al. [53] also pointed out that the differences in procyanidin levels begins when beans are obtained and preliminary processed. Despite the use of raw cocoa material change from a single batch, beans devoid of cocoa liquor and outer membranes were characterized by higher total procyanidin content, with a better average than beans traditionally obtained via splitting cocoa fruit and removing cocoa liquor. Regardless of which model was used, including various combinations of fermentation methods (spontaneous or induced) and bean drying (hot air or sun), as well as sample size, a significant decrease in all procyanidin polymers was observed. According to the results of this study, most of the procyanidin loss was attributed to drying, which, in light of the presented data, was the critical step for procyanidin loss at a summed level of about 65–70%.

### 2.2. Analysis of the Content of Major Flavanoids in CMM

As shown in Table 1, the levels of individual flavan-3-ols varied significantly depending on the parameters of the conching process. According to Gttumukkala et al. [7], the presence of excipients such as sugar, milk fat, and vanilla did not interfere with the determination of catechin and epicatechin in chocolate. Nevertheless, it is important to mention the mechanisms by which milk proteins limit cocoa polyphenol availability, which have only been thoroughly investigated in the last decade. Gallo et al. [54] clearly indicated the role of β-lactoglobulin (whey milk protein) in the formation of stable complexes with epicatechin and catechin through covalent bonds. At the same time, the authors suggested that non-covalent bonds with milk casein proteins also reduce flavanol availability. According to Prawia and Barringer [55], the perceived quality of milk chocolate is affected by conching time, sucrose, lecithin, cocoa butter, and whole milk powder. Texture, on the other hand, is mostly influenced by conching, milk powder, lecithin, and cocoa butter.

#### 2.2.1. Catechins Content in CMM

**Figure 2:**
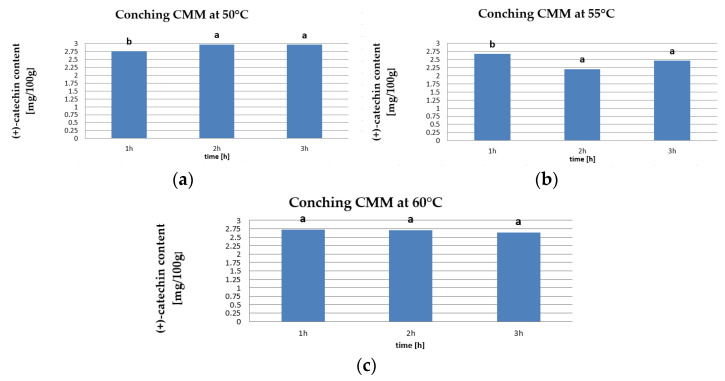
Catechin content in CMM at conching temperatures (**a**) 50 °C, (**b**) 55 °C, and (**c**) 60 °C (the same letters mean that there are no statistically significant differences between the values of this indicator at a confidence level of α = 0.05).

In the present study, (+)-catechin was determined at 2.2–2.97 mg/100 g. Based on the results of the analyses, the content of this compound during 3 h of conching at 50 °C increased by 7.3%. At 55 °C it decreased by 8% and at 60 °C it also decreased but only by 2.9% (Figure 2).

The obtained values of catechin content were subjected to statistical analysis and the Tukey HDS test. Values of this index were assigned to two homogeneous groups at 50 °C conching temperature and three homogeneous groups at 55 °C. At 60 °C, there were no significant differences between conching times. At a conching temperature of 50 °C, no significant differences were found in values between 2 and 3 h of conching. Żyżelewicz et al. [56] used the functionalization of cocoa solids from raw cocoa beans to study its effect on the content of bioactive components in chocolate. They determined the content of (+)-catechin at 2.15 mg/100 g in chocolate made only with the use of intermediates obtained from roasted beans and 1.87 mg/100 g in the product obtained from beans subjected only to fermentation and drying. Chocolates based on unroasted beans contained less catechins than those based on roasted beans. On the other hand, in a study of ready-made chocolates from the market, Żyżelewicz et al. [57] determined the catechin content in milk chocolates (cocoa mass content: 32–43%) to be 1.3–6.7 mg/100 g, which coincided with our results. In dessert chocolates (cocoa mass content: 60–64%), catechins were determined in amounts of 13.81–17.52 mg/100 g, while in dark chocolates they were 37.28 mg/100 g (chocolate with 74% cocoa mass content). Cooper et al. [58] found the content of catechin in milk chocolate to be in the range of 4–12 mg/100 g, while in dark chocolates it was much higher, i.e., 7–52 mg/100 g. Arts et al. [59] confirmed that the highest content of polyphenols was found in dark chocolate, with a total content of 61 mg of catechins/100 g of fresh edible mass, which is related to the content of non-fat components (a source of polyphenols).

#### 2.2.2. Epicatechins Content in CMM

**Figure 3:**
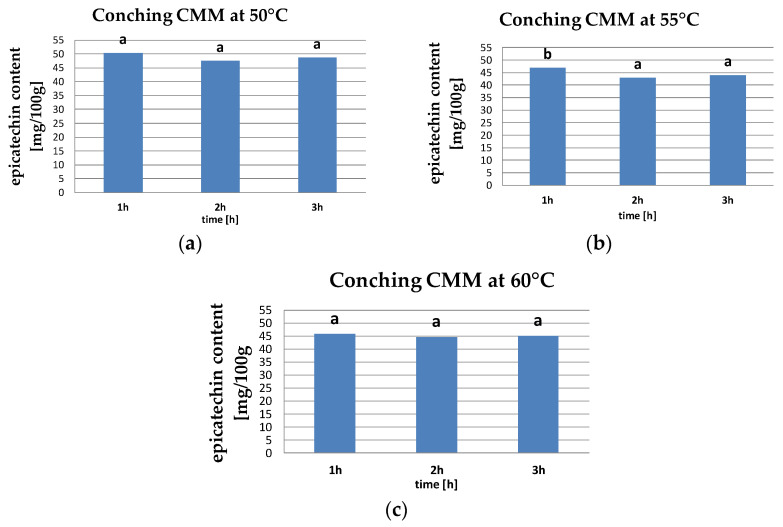
Epicatechin content in CMM at conching temperature of (**a**) 50 °C, (**b**) 55 °C, and (**c**) 60 °C (the same letters mean that there are no statistically significant differences between the values of this indicator at a confidence level of α = 0.05).

In our study, epicatechin content ranged from 42.86 to 50.36 mg/100 g. Our study showed that at conching temperatures of 50, 55, and 60 °C, the epicatechin content decreased from 1.5 to 6.1%, respectively, during 3 h of conching (Figure 3).

The obtained values of epicatechin content were statistically analyzed. On the basis of the Tukey HDS test, no significant effect was found for the CMM conching time at 50 and 60 °C. Only at 55 °C were the two homogeneous groups separated; however, no significant differences in the samples were conched for 2 and 3 h.

Żyżelewicz et al. [57] observed that the range of epicatechin content in milk chocolates (cocoa mass content: 32–43%) was between 5.91–18.8 mg/100 g, which was more than twice as low as our results. In all chocolates studied by these researchers, compounds belonging to the flavan-3-ols group were present at the very highest concentrations. The dark chocolate made from Ecuadorian cocoa beans contained 80% cocoa solids and proved to be the richest source of polyphenols. Epicatechin content was 280.59 mg/100 g. On the other hand, dessert chocolates contained 79.42–101.26 mg/100 g of epicatechin. Cooper et al. [58] found the epicatechin content of milk chocolate to be between 19–50 mg/100 g, while in dark chocolates the spread was between 7–194 mg/100 g. The upper limit of epicatechin content in milk chocolate was similar to our results. 

Alanon et al. [43] determined the epicatechin content in milk chocolate to be 8.5–63.7 mg/100 g, while in dark chocolate it ranged from about 3.8 to about 80.7 mg/100 g. Again, the upper range of epicatechin content was comparable to our results. The increasing importance of epicatechin as a component of therapeutic interest necessitated the development of highly sensitive and selective methods by Gttumukkala et al. [7]. A simple reversed-phase HPLC method was developed, and the results showed that the developed method was highly specific, accurate, and precise, and can determine catechin and epicatechin content in chocolate. Interestingly, branded chocolate (e.g., Lindt, Godiva Chocolatier) contained between 17.32 and 31.68 mg/100 g of epicatechin. Chocolates of Indian origin contained less epicatechin (only 3.6 mg/100 g) compared to brands available in the USA [7]. This is probably due to the beans used to make the cocoa liquor, as they came from different growing regions. They were also subjected to different processing in different parameter ranges. 

Langer et al. [60] noted that epicatechin was the largest fraction of all chocolate flavonoids. In milk chocolate (20% cocoa content), it was only 10 mg/100 g, while in dark chocolates (bitter) the content of this compound ranged between 29.8–269.7 mg/100 g. In addition, the researchers proved that the concentration of epicatechin can indicate the total content of flavanols and procyanidin, while the percentage of cocoa mass cannot be a clear indicator of (−)-epicatechin content. Šeremet et al. [61] determined the epicatechin level for ruby chocolate (new to the chocolate market) to be between 90–102 mg/100 g. The content of the determined flavanol could be due not only to the different fermentation method of the beans but also to the percentage of individual raw materials, mainly sugar. In our study, the epi/cat ratio for CMM at a conching temperature of 50 °C ranged from 16.4 to 18.1, i.e., it decreased slightly during conching. At 55 °C, the ratio increased from 17.4 to 17.86, while at 60 °C the ratio remained between 16.81 and 17.0. 

In this study, a temperature difference of +/− 5 °C and a processing time of +/− 5 min were used. Differences in the ratio of catechin and epicatechin content were shown, but the differences were not unambiguous. Conducting the conching process for 1 h at varying temperature ranges indicated a reduction of the calculated index, which confirmed the epimerization of epicatechin to catechin. Statistical analysis distinguished three homogeneous groups and a *p*-value = 0 for both the influence of the variability of process time and temperature. The smallest differences were found for conched masses at 60 °C in various points throughout the process. The results were classified into one homogeneous group with a *p*-value = 0.2016, which indicates that with these conching parameters there were no significant changes in the configuration of the phenols determined. Miller et al. [62] found that the epicatechin to catechin ratio varied between 1.0 for a single liquor sample to 5.1 for a milk chocolate sample. The authors stated that there was a wide range in the ratio of monomeric composition of tested products. Many studies [28,29,30] have indicated that the ratio epi/cat tends to decrease when high temperatures are applied because of the degradation of epicatechin relative to catechin, among other things. In our study, the temperatures used during the conching process were up to 60 °C. According to Hurst et al. [32], it is the high temperature that can trigger the epimerization of flavanol to (−)-catechin and (+)-catechin to (+)-epicatechin.

#### 2.2.3. Procyanidin B2 Content in CMM

**Figure 4:**
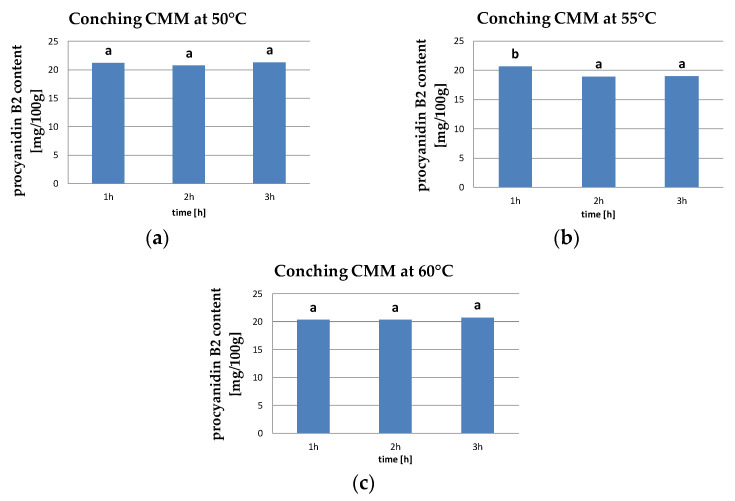
Procyanidin B2 content in CMM at conching temperature of (**a**) 50 °C, (**b**) 55 °C, and (**c**) 60 °C (the same letters mean that there are no statistically significant differences between the analyzed products at a confidence level of α = 0.05).

In our results, the range of procyanidin B2 content in CMM was between 18.93–21.28 mg/100 g. Our study showed that after 3 h of conching at 50 and 60 °C, procyanidin content increased by 0.3 and 2.1%, respectively, while at 55 °C its level decreased by 8% during 3 h conching (Figure 4).

There was no significant effect of conching time on the procyanidin B2 content in CMM at 50 and 60 °C. Only at the conching temperature of 55 °C did two homogeneous groups form, yet no significant differences were found between 2 and 3 h conching times.

di Mattia et al. [23] analyzed the effect of two conching processes: long-term conching (LTC) and short-term conching (STC) on the content of bioactive compounds and their activity in chocolate. The procyanidin content and type were strongly influenced by different processing conditions. After conching, STC samples showed a higher amount of monomers compared to LTC, which resulted in more polymerization and confirmed the presence of polymers in the studied samples. Researchers concluded that procyanidin content and type were affected by different processing conditions. On the other hand, in a study by Żyżelewicz et al. [57], the range of procyanidin B2 content in milk chocolates (cocoa mass content: 32–43%) were found to be at a similar level (3.94–24.7 mg/100 g) as ours. Levels of procyanidin B2 in chocolate samples developed by Żyżelewicz et al. [56] based on roasted and unroasted beans were much higher than ours, ranging from 191 to 322 g/100 g of product. Comparing the level of procyanidin B2 in the sample, for which the calculated cocoa solids content was 40.0%, to the results obtained in our study, it can be noted that it was 10 times higher. The reason for this situation could be that the researchers used a specially prepared blend of beans of the Criollo and Forastero varieties. It is estimated that the content of polyphenols in the beans of the Criollo variety is about two-thirds the amount present in the Forastero variety. However, Criollo cocoa beans show higher levels of procyanidin content than Forastero and Trinitario cocoa [27,52,63]. The significantly higher levels of procyanidin B2 in raw chocolates may also have been influenced by the conching process, which in this case is designed to remove undesirable volatile compounds, such as acetic acid. This reduced during the traditional manufacturing method at the roasting stage. di Mattia et al. [23] also reported that a higher amount of procyanidin monomers formed when conching cocoa mass at a high temperature in a short amount of time, while the polymerization process of these compounds became more dynamical during the operation at low temperatures for longer periods.

Gültekin-Özgüven et al. [64], in a three-factor analysis of bean processing, considered the temperature of the roasting process, alkali concentration in the alkalization process (ultimately the pH of their solutions), and the conching temperature of cocoa mass. They found that all factors contributed to a decrease in procyanidin content in the dessert chocolate prepared for the test. However, the first two factors were considered the most important. Significant losses of procyanidin B2 were observed when the pH increased to 8 in conjunction with the roasting temperature increasing to 147 °C. The contents of procyanidin B2 in the reprocessed chocolate samples contained about 43% cocoa solids and ranged from nearly 73 to about 161 mg per 100 g sample. Different trends were obtained by Šeremet et al. [61], who determined the content of procyanidins, expressed as cyanidinium chloride equivalents, in different types of chocolate. The researchers determined 10 mg of procyanidin in 100 g of ruby chocolate and 80 and 56 mg/100 g in dark chocolate (72.0% cocoa mass) and dessert chocolate (38.0% cocoa mass), respectively. The reason for such a distribution of procyanidins in the samples may be both the starting material itself, the different origin and method of making chocolate, as well as the applied methodology for the determination of the selected flavanol groups. According to Langer et al. [60], the amount and integrity of procyanidins during production was determined (degraded) due to oxidation and alkalization. The authors pointed out that the content of cocoa ingredients in chocolates did not always reflect flavonoid levels. However, increasingly, a high proportion of phenolic compounds is used commercially to reflect chocolate quality.

### 2.3. Rheological Properties of CMM

Chocolate’s rheological properties, based on its acceptance by consumers, are largely determined by the ingredients and their proportions used in recipes. For example, the smooth texture and taste of milk chocolate are determined by the addition of milk powder [65].

Chocolate’s rheological properties are influenced by processing (e.g., refining, conching, tempering), as well as the raw material composition and properties (e.g., particle size distribution, amount of fat, amount and type of emulsifiers added) [66,67,68]. Rheological properties not only determine the efficiency of processes including mixing and transport (pumping) but they also play a key role in processing [26]. One of the important parameters of chocolate mass is viscosity, which is usually expressed via the Casson model, whose reference values for dark chocolate have been defined as ranging between 2.1–3.9 Pa·s [69].

Conching conditions show an interaction between time and temperature of the treatment. Higher temperatures allow for shorter processing times. The length of time required varies depending on the components of the conching mass and the origin of some of them (e.g., cocoa beans). Increasing the cocoa fat content in chocolate reduces viscosity and yield stress [70].

In our study, the viscosity of CMM was ranged between 1.85–2.8 Pa·s (Table 3). During conching at 50 °C for 3 h, this value decreased by more than 15% (to 2.21 Pa·s), which was similar as what happened after the process at 55 °C (2.42 Pa·s). In contrast, the masses conched at 60 °C for 1 h were characterized by the viscosity of 1.94 Pa·s, which decreased after 3 h by about 5% (1.85 Pa·s). Significant influence of conching time on Casson viscosity of CMM was demonstrated. The Tukey HDS test showed significant differences for the conching temperature of 50 °C, creating three homogeneous groups that depended on conching time. Statistical analysis of Casson viscosity at 55 °C created two homogeneous groups but there were significant differences only between the conching times of 1 and 3 h. No significant differences in Casson viscosity were found between conching time at 60 °C (one homogeneous group). Regression analysis with 95% confidence interval showed a high negative correlation (−0.67) between Casson viscosity and conching time at 50 °C. A similar correlation (−0.93) was found at a 55 °C conching temperature, while for 60 °C the correlation was only −0.57.

A similar tendency was noticed when measuring yield stress, which ranged between 3.54–5.28 Pa for all investigated CMM samples (Table 3). At the conching temperature of 50 °C, yield stress decreased by almost 30% (3.75 Pa), whereas at 55 °C it decreased by more than 15% (3.93 Pa). Further, at 60 °C, the yield stress decreased by less than 9% (3.54 Pa). Statistical analysis showed a significant effect of the time between the use of 1 and 3, and 2 and 3 h on yield stress at conching temperatures 50 and 55 °C. However, at 60 °C, no significant differences were found.

Regression analysis showed that there was a high negative correlation (−0.92) between yield stress and conching time at 50 °C, which was a similar correlation as −0.91 and the 55 °C conching temperature. Further, while at 60 °C, the correlation was the lowest, at −0.63.

Maheshwari and Reddy [71] reported that for milk chocolate with added kokum fat the Casson viscosity ranged between 1.51–1.6 Pa·s. Interestingly, the control sample used similar proportions as our study (sugar content 41%; milk powder content 18%) and conching was carried out for 3 h at 50–55 °C. The determined viscosity was 1.63 Pa·s, which was twice as low as our study. In contrast, Toker et al. [72] determined the Casson viscosity of CMM samples in the range of 1.83–2.31 Pa·s, which was comparable to our results. According to Aidoo et al. [73], the Casson viscosity parameter was strongly influenced by the dry conching time. The authors [73] showed that chocolate samples dry conched for 10 min resulted in higher Casson viscosity than samples dry conched for 15 min, regardless of the wet conching temperature. According to the researchers, this may be related to the formation of a fatty envelope around the ingredient particles, which increased the particle size distribution and resulted in a lower Casson viscosity. In our case, dry conching was used for 10 min, which may have had a positive effect on this parameter. The authors also noted that during wet conching, temperature had very little effect on rheological properties and no definite trend was observed.

Variations in conching temperature and time can change viscosity and the final texture of the chocolate. This depends on the ingredients used, as well as the water content of the raw materials. Bolenz et al. [12] used xylitol as a sweetener in their study. They showed an increase in mass viscosity after conching. They found that water contained in milk was released and bound (absorbed) by xylitol. If some of the water was not absorbed by the highly hygroscopic sweetener, the viscosity of the whole mass increased.

On the other hand, a study by Pirouzian et al. [74] evaluated the effect of sugar substitutes on the rheological characteristics of milk chocolate using a simplex-crate mixture design. For this purpose, two bulking agents (maltitol and xylitol) were used at different levels (0–100%) and 10 formulations were tested to find the optimal levels. It was found that the compounded milk chocolate showed shear thinning behavior. Chocolate formulations containing the highest maltitol substitution gave similar rheological properties compared to the control sample, and therefore may be a good alternative to the basic composition. The results showed that chocolate combinations containing 87.8% maltitol and 12.2% xylitol had optimum concentrations that gave them the most acceptable rheological properties. Viscosity ranged between 1.6–2.6 Pa*s, which was consistent with our data. Medina-Mendoza et al. [75] determined the viscosity limit value in dark chocolate partially enriched with Sacha Inchi oil ranged between 1.6–1.81 Pa*s, which was close to the lower limits of the range obtained in the present study.

### 2.4. Sensor Analysis of CMM

The CMM solids samples showed high sensory acceptance with respect to color and aroma. On the other hand, sufficient and good quality levels were achieved by such quality characteristics as texture (i.e., hardness and smoothness), product breakthrough, and taste.

Due to the fact that the CMM were not subjected to the tempering and molding processes, the following parameters were omitted in the sensory evaluation: shape and the appearance of upper and lower surfaces.

Every evaluated MCC sample had the color characteristics of milk chocolate. All samples scored above 4 in this discriminant, indicating that the color was appropriate, clear, and uniform. Table 4 presents the sensory evaluation details.

The tested CMM were mostly free from foreign odors. The perceptible aroma was characteristic of this product group. The team members rated the highest aroma of CMM to be conched at 50 °C. The prolongation of conching time decreased the intensity of the chocolate aroma, which was confirmed by lower marks. Aroma compounds belonged to the group of volatile compounds; therefore, under the influence of temperature they evaporated, which affected the sensory experience. In addition, undesirable compounds can form under the influence of temperature (including, for example, Maillard, which in turn can affect the sensing of uncharacteristic, foreign aromas).

The turn of the CMM, defined as matter with no porosity, in all CMM tested samples were rated at a tolerable quality level. Team members found the breakthrough to be correct in each of the CMM conched for 3 h, but in CMM it was conched for only 1 h. Lighter spots were noticeable on the cross-section, which contributed to the lower score. During the evaluation of consistency, the team members considered such distinctions as smoothness (understood as the impalpability of a component’s when spreading the sample in the mouth) and hardness (the resistance of the sample when chewing). Due to the omission of the rolling process, the particle size in the tested CMM could result in the perception of lumps. Therefore, smoothness and breakthrough were rated quite low. In the case of CMM conched at 50 °C, these characteristics were evaluated as having an undesirable quality, resulting from the sensation of product particles when the sample was spread in the mouth. In the taste evaluation, the team members considered the overall sensory impression. After averaging the evaluations, the CMM conched at 55 °C were found to be the best. The evaluators found that these were the samples with the most pleasant chocolate flavor and that they melted in the mouth. The CMM used a typical sugar content (45%) for commercial chocolate. It is likely that this high sugar content suppressed the bitterness of the cocoa aftertaste, which may have occurred due to the use of cocoa liquor based on unroasted cocoa beans.

Oberrauter et al. [76] found phenolic compounds to be key inducers of bitter taste and astringency, which potentially limits consumer acceptance of chocolate with higher cocoa content. In our case, the cocoa solid content was 16% and the addition of milk at 18% significantly mitigated or eliminated the bitter aftertaste.

Jovanka et al. [77] showed the sensory properties of nine milk chocolate preparations that differed in sucrose (400, 475, or 550 g kg^−1^) and cocoa fat (280, 320, or 360 g kg^−1^) contents were investigated. It was shown that samples with low sugar content were more bitter and grainy, with a noticeable roasting aftertaste, while the samples with high sugar content were characterized as having a more intense milk flavor, vanilla/caramel flavor, hard texture, and more sweetness. In our analyses, we used high sugar content (45%) to contribute to improving the taste and aroma of the conched mass and thus meeting the expectations of consumers and panelists.

Guinard et al. [78] showed that different levels of fat and sugar resulted in very large differences in the sensory properties of milk chocolate. Milk chocolate varied in sucrose content (400, 475, or 550 g kg^−1^) and in cocoa fat content (280, 320, or 360 g kg^−1^). Samples with high fat content were rated lower than samples with low fat content. It should be noted that the limited range of cocoa fat concentrations in samples (28 to 36%) may have partially accounted for the panel’s apparent inability to discern differences in fat content in the chocolate.

Drewnowski and Schwartz [79] also found that sugar masks the sensory evaluation of fat in some solid foods. High levels of fat make chocolate less sticky, while while milk powder has the opposite effect. An interesting study was conducted by Biancolillo et al. [80], who aimed to develop an analytical methodology to correlate the sensory poles of chocolate with their chemical properties and ultimately with the cocoa beans used in its preparation. Trained panelists examined several chocolate samples and divided them into four groups (characterized by 36 different descriptors) that can be attributed to chocolate flavor. They found that in the case of chocolate, sensory perception related more to volatile composition, while sensory perception of cocoa beans depended more on polyphenol and organic acid content.

The different taste perception of different cocoa-based products may be related to the profile of volatile compounds in these products, which was also analyzed by Liu et al. [81], Rottiers et al. [82], and Hinneh et al. [83].

Mezo-Solís et al. [84] Lê and Husson [85], and Leite et al. [86] observed that the attributes of milk aroma, cocoa aroma, bitter, brown, and chocolate smell make it possible to discriminate against chocolate. Results from the panelists showed consensus in five (Cocoa-F, Cocoa-A, Chocolate-A, Fat-A, and Sweet-AT) out of eight evaluated attributes.

Thamke et al. [87] found that the key sensory attributes of industrial chocolate are sweet and cocoa flavor, which is also consistent with our results.

Aidoo et al. [73] found that chocolate made from vegetable “milk” was acceptable to consumers because it was rated highly in all critical sensory attributes of taste, mouth feel, good appetizer, and sweet taste. Yet to achieve this acceptability, the chocolate recipe must contain no more than 18% cocoa solids and no more than 30% sugar. These two ingredients have a strong influence on chocolate hardness, flavor, and graininess in the mouth, which are critical to consumer acceptance of chocolate. In our case, it is likely that the use of more sugar resulted in the hardness of samples being at a sufficient level with a slight indication of “not very hard” for samples conched at 50 °C.

On the other hand, Lanza et al. [88] studied the sensory properties of chocolate with the presence of larger sugar crystals and a sandy texture, resulting from a particular form of production. Despite its diversity, chocolate according to the panelists did not lose two sensory descriptors typical of chocolate: melting and cocoa taste. Misnawi et al. [89] studied the changes in the ability of polyphenols to induce astringency during the fermentation and roasting of cocoa beans. The results indicated that polyphenols were positively correlated with astringency and bitterness. The presence of different levels of polyphenols in the products may have been due to the fermentation and roasting conditions. However, according to the authors, polyphenols are essential for the sensory properties of cocoa. In our case, the use of cocoa liquor from unroasted beans resulted in major polyphenol content, at least three times higher than that of cocoa liquor from roasted beans. However, the use of milk meant that the typical bitter aftertaste was not perceptible in the tested samples.

## 3. Materials and Methods

### 3.1. Research Material

Cocoa liquor, cocoa fat, milk powder, sugar, and lecithin were used to produce chocolate milk mass (CMM).

The cocoa liquor was made from unroasted cocoa beans sourced from organic farms in Peru. All other raw materials were obtained from the chocolate product manufacturer. The dry ingredients of CMM were prepared by blending the following ingredients:

- Cocoa liquor—16%
- Cocoa fat—20%
- Sugar—45%
- Milk powder—18%
- Lecithin—0.5%

### 3.2. Technological Process

CMM were prepared from ingredients weighed and mixed at controlled tempera-ture and time in a Termomix machine (Vorweck, Wuppertal, Germany). Before the chocolate mass were properly prepared, a trial series was carried out. They were checked among others temperature indications on the device using an external thermometer. In each case, the deviation of the temperature measurement was not greater than ±0.5 °C. The first step was to liquefy the weighed portion of cocoa butter at 45 °C (the liquefied fat was poured into another vessel). Next, the cocoa liquor was liquefied at 50 °C and sugar, milk powder, and 10% cocoa fat were added. The ingredients were mixed for 10 min at a constant temperature of 45 °C. Then, the remaining cocoa fat and lecithin were added. Conching of the obtained mass was carried out at 50, 55, and 60 °C for 1, 2, and 3 h (Table 5). The basic conching parameters were indicated by the confectionery company, while their modifications were chosen on the basis of preliminary tests.

### 3.3. Analytical Methods

#### 3.3.1. The Determination of Flavanols Using High Performance Liquid Chromatography

For chromatographic quantification of individual flavanols in the samples, the following were used: flavanol standards—(+)-catechin hydrate (≥98%), (−)-epicatechin (≥98%), and procyanidin B2 (≥90%); acetic acid AR, methanol AR, and sodium carbonate (≥99.5%). All substances were purchased from Sigma-Aldrich. Steps of sample preparation for chromatographic separation included the CMM sample (approximately 5 g), which was crushed and weighed into flasks to the nearest 0.0001 g. Next, 150 mL of methanol (pure and without dilution) extraction was carried out in a water bath for 20 min at 30 °C. The obtained extracts were filtered through a paper filter and then evaporated on a vacuum evaporator to a final volume of about 100 mL. The resulting solution was filtered through a 0.45 μm pore diameter membrane filter and subjected to chromatographic determination. Detection of compounds was carried out via a high-performance liquid chromatography (HPLC) (Agilent 1200) using a diode array detector. A reversed phase column (Zorbax, C18, 5 μm, 250 × 4.6 mm) was used and its temperature was maintained at 30 °C.

A Diode Array Detector Agilent 1200 with a deuterium lamp flow cell and a 10 mm optical path was used. Specifications are as follows: wavelength range: 190–640 nm; wavelength setting error: ±1 nm, self-calibration; spectral slit width: 4 nm; simultaneous signal recording: up to 8 wavelengths; sampling rate: 80 Hz; transient noise: ± 0.6 × 10^−6^ AU at 230 nm; drift: 5 × 10^−4^ AU/h at 230 nm).

The mobile phase was prepared as follows. The following eluents were used: solution A—0.1 mL of acetic acid was dissolved in 900 mL of water and made up to 1000 mL; then, the solution was filtered through a 0.45 µm membrane filter and degassed in a sonicator for 3 min. Solution B was pure methanol. The elution of the compounds was a gradient from 0.01 min of separation of 11% solution B, then from 30 min of 25% solution B, from 35 to 39 min of 100% solution B, and then from 40 to 50 min of 11% solution B. The mobile phase flow rate was 0.6 mL/min and the extract injection volume was 5 µL. The extracts were detected and analyzed at an analytical wavelength of λ = 280 nm. Calculations were performed using the absolute calibration method, which considered the purity of the standard substances. Stock solutions of (+)-catechin-hydrate, (−)-epicatechin, and procyanidin B2, at concentrations of 1 mg/mL each, were prepared by dissolving the standards in methanol. Calibration curves were plotted based on the area under the peak corresponding to the detection of each compound at seven different concentration levels (dilutions of stock solution in methanol): 0; 2.5; 5; 10; 15; 20; and 30 µg/mL in six replicates. The curve fit to the empirical points was determined via the linear coefficient of determination R^2^, which was R^2^ > 0.998 for all analyses. Retention times for individual flavanols under the conditions described above were 16.1 min for (+)-catechin, 18.7 min for procyanidin B2, and 24.2 min for (−)-epicatechin.

#### 3.3.2. The Determination of the Protein Content

The determination of the protein content was carried out in milk powder and cocoa liquor samples. The protein content was determined using the Kjeldahl method. The principle of the method was to digest the samples with concentrated sulfuric acid (VI) in the presence of a catalyst (selenium-copper mixture) in a Buchi 426 Dugestion unit. Under these conditions, protein nitrogen was converted to ammonium ion, which after alkalization was distilled as ammonia and bound with excess boric acid in Buchi B-316 distillation unit. The ammonia solution was determined using potentiometric titration with 0.1 N hydrochloric acid standard solution. The nitrogen content of the sample was calculated from the volume of 0.1 N standard hydrochloric acid solution used for titration, knowing that 1 cm^3^ of 0.1 N hydrochloric acid solution corresponds to 0.0014 g of nitrogen. Nitrogen was converted to protein using a multiplier calculated from the average nitrogen content of the proteins, which is 16% (100:16 = 6.25). The determination was carried out in three consecutive replicates.

#### 3.3.3. Rheological Properties

Rheological measurements were carried out in a shear rate-controlled rheometer HAAKE MARS 40 (Thermo Scientific, Germany) equipped with coaxial cylinders (CC25 DIN Ti measuring geometry) [90]. Before taking measurements, sample were heated at 50 °C for 60 min for melting. The measurement was set up according to the ICA analytical method 46 (International Confectionary Association), with an additional waiting time of 5 min at 40 °C before the measurement started. Yield stress was measured at 40 °C as a function of increasing the shear rate from 2 to 50 Pa within 180 s, then was kept constant for 60 s at 50 Pa. After that, it decreased from 50 to 2 Pa within 180 s. The obtained flow curves were fitted with Casson model using the HAAKE RheoWin 4.7 software to obtain yield stress and viscosity.

#### 3.3.4. Sensory Evaluation

Sensory evaluation was performed with a 15-member team using a standardized method based on PN-98/A-88032 [91]. The following quality characteristics were evaluated: color, hardness, smoothness, turn, taste, and flavor. The evaluators had at their disposal the appendix of the Polish Standard, containing the characteristics of the individual distinguishing factors according to the range of points awarded.

#### 3.3.5. Statistical Analysis

Excel (Microsoft Office, 2019) was used to calculate the mean values of the obtained results, standard deviations, and to create graphs. Statistical analysis of the results obtained, correlation, and significant tests for differences between the test samples were carried out using Statistica 13.0 statistical software, Pearson’s correlation coefficient, and Tukey HDS test with a *p* < 0.05 level of significance, respectively.

## 4. Conclusions

As expected, epicatechin was the dominant flavan-3-ol in all analyzed CMMs. Among the dominant flavan3-ols, the obtained and analyzed samples contained the least amount of catechins, which was consistent with the literature. The content of phenolic compounds is primarily related to the composition of raw material. Phenols are in the fat-free components of cocoa beans; it is their amount in the product composition that determines the content of determined compounds. Moreover, the variety, region, and cultivation conditions of cocoa beans influence the content of phenolic compounds in the raw material, as well as indirectly in the final product. Manufacturing processes and their parameters—mainly temperature and time—are another important aspect. Basic rheological parameters, such as viscosity and liquid limit, were within ranges typical for milk chocolate reported by other researchers. We demonstrated the influence of both the temperature and conching time on rheological properties for chocolate milk mass. After 3 h of conching at 50 °C, viscosity dropped by about 15%. The increase in conching temperature caused smaller differences in viscosity values, respectively, at 55 °C by about 13.5%, and at 60 °C by about 5%. Conching at 50 °C also caused the greatest decrease in yield stress (by about 30%). The conched samples at the temperature of 55 °C were characterized by the determined parameter (about 15% lower), while at 60 °C it resulted in lowering the value of the yield stress by about 9%. In summary, the greatest influence on rheological properties were expressed in viscosity and yield stress. This was shown in a CMM conched mass at 50 °C, which demonstrated high sensory acceptance with respect to color and aroma. Sufficient quality was reached, specifically vis-à-vis consistency (hardness and smoothness), product breakthrough, and taste.

## Figures and Tables

**Table 1:** Content of (+)-catechins, epicatechins and procyanidins B2 in CMM (the abbreviations used in the graph are described in Section 3.2).

Sample	(+)-Catechin [mg/100 g]	Epicatechin [mg/100 g]	Procyanidin B2 [mg/100 g]	epi/cat
C501h	2.77 ± 0.15	50.36 ± 0.23	21.22 ± 0.05	18.21
C502h	2.97 ± 0.02	47.49 ± 0.34	20.73 ± 0.19	15.97
C503h	2.97 ± 0.13	48.71 ± 0.21	21.28 ± 0.14	16.41
C551h	2.68 ± 0.11	46.81 ± 0.09	20.64 ± 0.34	17.48
C552h	2.21 ± 0.05	42.87 ± 0.17	18.93 ± 0.26	19.42
C553h	2.46 ± 0.09	43.94 ± 0.07	18.99 ± 0.21	17.84
C601h	2.72 ± 0.07	45.74 ± 0.18	20.34 ± 0.04	16.79
C602h	2.7 ± 0.06	44.69 ± 0.23	20.33 ± 0.08	16.56
C603h	2.65 ± 0.14	45.07 ± 0.34	20.76 ± 0.32	17.04

**Table 2:** Content of major flavonoids and proteins found in raw cocoa materials used for production.

Raw Material Name	Content of (+)-Catechin [mg/100 g]	Content of Epicatechin [mg/100 g]	Content of Procyanidin B2 [mg/100 g]	Protein Content [%]
Cocoa beans (unroasted)	13.56	261.34	107.13	nb
Cocoa liquor (based on unroasted beans)	11.61	226.65	93.0	13.82

**Table 3:** Rheological properties of CMM conched under different times and temperatures (the same letter means that there are no statistically significant differences between the analyzed products at a confidence level of α = 0.05; the abbreviations used in the graph are described in Section 3.2).

Sample	Yield Stress (Pa)	Viscosity (Pa·s)
C501h	5.28 ± 0.25 ^a^	2.61 ± 0.34 ^b^
C502h	4.83 ± 0.34 ^a^	2.78 ± 0.02 ^c^
C503h	3.75 ± 0.78 ^b^	2.21 ± 0.04 ^a^
C551h	4.64 ± 0.65 ^a^	2.80 ± 0.23 ^b^
C552h	4.44 ± 0.23 ^a^	2.61 ± 0.12 ^a,b^
C553h	3.93 ± 0.13 ^b^	2.42 ± 0.23 ^a^
C601h	3.89 ± 0.45 ^a^	1.95 ± 0.13 ^a^
C602h	3.55 ± 0.65 ^a^	1.95 ± 0.32 ^a^
C603h	3.54 ± 0.54 ^a^	1.85 ± 0.14 ^a^

**Table 4:** Overall sensory evaluation of the tested CMM using the five-point method (the abbreviations used in the graph are described in Section 3.2).

Sample	Color	Hardness	Smoothness	Turn	Flavor	Taste
C501h	4.17	2.99	2.7	2.9	4.8	3.15
C502h	4.133	3.27	2.9	2.7	4.7	3.34
C503h	4.57	3.075	3.1	2.8	4.6	3.65
C551h	3.9	2.85	2.95	3.5	3.9	4.55
C552h	4.3	3.05	3.1	3.65	4.15	4.2
C553h	4.1	3	3.35	3.9	4.25	3.9
C601h	4.15	3.69	3.35	3.7	4.45	3.75
C602h	4.35	3.8	3.48	3.85	3.9	3.55
C603h	4.65	3.88	3.89	4.15	4.2	4.1

**Table 5:** Designation (codes) of CMM and conching parameters.

Code Sample	Conching Parameters [Temperature; Time]
C501h	Temp. 50 °C; 1 h
C502h	Temp. 50 °C; 2 h
C503h	Temp. 50 °C; 3 h
C551h	Temp. 55 °C; 1 h
C552h	Temp. 55 °C; 2 h
C553h	Temp. 55 °C; 3 h
C601h	Temp. 60 °C; 1 h
C602h	Temp. 60 °C; 2 h
C603h	Temp. 60 °C; 3 h

## Data Availability

Not applicable.

## Sample Availability

Samples available from the corresponding author.
